# *Pharmaceuticals and Environment*: a web-based decision support for considering environmental aspects of medicines in use

**DOI:** 10.1007/s00228-020-02885-1

**Published:** 2020-05-09

**Authors:** Helena Ramström, Siv Martini, Johanna Borgendahl, Marlene Ågerstrand, Gerd Lärfars, Marie-Louise Ovesjö

**Affiliations:** 1 Health and Medical Care Administration, Region Stockholm, Box 6909, SE-102 39 Stockholm, Sweden; 2 Formerly at Health and Medical Care Administration, Region Stockholm, Stockholm, Sweden; 3 Sustainability Department, Regional Executive Office, Region Stockholm, Stockholm, Sweden; 4 Department of Environmental Science, Stockholm University, Stockholm, Sweden; 5 Division of Clinical Pharmacology, Department of Laboratory Medicine, Karolinska Institutet, Stockholm, Sweden; 6 Department of Quality, Development, MT and IT, Södersjukhuset, Stockholm, Sweden

**Keywords:** Pharmaceuticals, Environmental classification, Environmental hazard assessments, Environmental risk assessment, Decision support

## Abstract

**Purpose:**

The database *Pharmaceuticals and Environment* is a non-commercial, freely available web-based decision support presenting compiled environmental information for pharmaceutical substances. It was developed by Region Stockholm and launched in 2016 at janusinfo.se. The purpose of this paper is to present the database, report on its current use, and reflect on lessons learned from developing and managing the database.

**Methods:**

A standard operating procedure describes the work and content of the database, e.g., how information is retrieved, processed, and presented. Google Analytics was used for metrics. Issues related to the database have been discussed and handled by a reference group. The experiences from this work are presented.

**Results:**

The database contains environmental hazard and risk information, primarily gathered from regulatory authorities and pharmaceutical companies. There are also assessments comparing substances within some groups of pharmaceuticals. The database is used by the Swedish Drug and Therapeutics Committees to include environmental aspects when recommending pharmaceuticals for health care providers. Page views show that users primarily look for information on commonly used substances, e.g., diclofenac and paracetamol/acetaminophen. Major problems for the development of the database are lack of data, lack of transparency, and discrepancies in the available environmental information.

**Conclusion:**

In the absence of an adequate decision support produced by the regulatory authorities, we find the database *Pharmaceuticals and Environment* to be useful for Swedish Drug and Therapeutics Committees and health care providers, and it is our belief that the information can be valuable also in other settings.

## Introduction

The use of pharmaceuticals is likely to continue to increase due to a growing aging population with chronic diseases, and access to new pharmaceuticals [1–3]. This is positive in terms of improved  health for the treated individuals but at the same time poses an increased risk to the environment. Studies have already shown that chronic exposure to low concentrations of ethinylestradiol leads to feminization of male fish and that levonorgestrel can bioconcentrate in fish and disrupt oogenesis in frogs [4–6]. The massive decline in vulture populations in the Indian subcontinent in the 1990s was caused by diclofenac poisoning [7], and behavioral changes in fish have been shown in studies with oxazepam and citalopram [8, 9]. In addition, antimicrobial resistance is an increasing problem and poses a serious threat to global health and antibiotic residues in the environment can be a contributing factor to this problem [10, 11]. Furthermore, high levels of pharmaceuticals in the effluent from the manufacturing process can cause local environmental problems [12].

Region Stockholm, the provider of most of the health care in Stockholm county, has been committed to reduce the environmental impact of pharmaceuticals since the beginning of the 2000s [13]. This work has included analyses of water samples for active pharmaceutical ingredients (APIs) and information to the public to increase the awareness of environmental effects of pharmaceuticals. To be able to focus management measures on the most harmful substances, environmental information on APIs was needed and therefore the work on gathering information began in 2001 [13]. The environmental information, which was originally presented in a printed brochure and on a webpage, is now available in the public database *Pharmaceuticals and Environment* at janusinfo.se in a Swedish and an English version [14]. The aim of the database is to present information about environmental hazard and risk associated with APIs for human use on the Swedish market.

In order to be an adequate tool for risk assessors and decision-makers, a database needs to provide accurate, updated, and easily accessible information that is adapted for the intended users [15]. There are other databases with the purpose of communicating research results and environmental information, thereby facilitating decision-making. LIF, the trade association for the research-based pharmaceutical industry in Sweden, presents environmental information for some of the medicinal products on the Swedish market at fass.se [16]. The European Medicines Agency (EMA) provides environmental risk assessments for medicinal products approved after 2006 in the European Public Assessment Reports (EPARs) at EMA’s website [17]. The WikiPharma database and the NORMAN Ecotoxicology Database both provide ecotoxicity data [15, 18]. The WikiPharma database was one of the outcomes of the Swedish MistraPharma research program during 2008–2015 which generated new data, e.g., identified pharmaceutical substances that pose risks in the aquatic environment, and issued recommendations for improved environmental risk assessments of human pharmaceuticals [19]. The ETOX database from the German Environmental Agency provides ecotoxicology data as well as information on various national and international environmental quality guidelines and limit values [20]. In the iPiE database, pharmaceutical companies present information on properties, environmental fate characteristics, and ecotoxicity of pharmaceuticals [21]. The US Environmental Protection Agency has developed the database ECOTOX which also contains data from ecotoxicity studies for pharmaceuticals [22]. The number of databases indicates that there is a need for decision support in this field, and that users may have different needs, hence the variety in focus and structure of the databases.

The purpose of this paper is to present the database *Pharmaceuticals and Environment*, report on its current use, and reflect on lessons learned from developing and managing a database intended as decision support.

## Method

### Developing the database

In Sweden, classification of environmental hazard of pharmaceuticals, presented per API, was initiated in 2001 by Region Stockholm and Apoteket AB (public owner of all pharmacies in Sweden at that time) [13]. In 2005, the work was jointly developed to include an environmental risk assessment by Region Stockholm, Apoteket AB, the Swedish Medical Products Agency, LIF, and the Swedish Association of Local Authorities and Regions [13]. The classification focused on environmental hazard and risk associated with APIs for human use on the Swedish market. This work resulted in the environmental information presented per medicinal product at fass.se. However, having the information presented per product was not suitable for the needs of Region Stockholm, and therefore the initial work with information presented per API was continued by Region Stockholm. Originally, this information was presented in a printed brochure and on a webpage. In 2016, the information handled in Excel was transferred to a Structured Query Language (SQL) database and in 2019 to a Content Management System (CMS) database, the same one used by janusinfo.se.

The database *Pharmaceuticals and Environment* gathers publicly available information about environmental hazard and risk. Some of the assessments have been generated by Region Stockholm or through academic experts. The work procedures and content of the database is based on a standard operating procedure for internal use. The document describes how environmental information is retrieved, processed, and presented; provides standard texts; suggests how to proceed if data are missing or incomplete; and suggests reference literature that can be used.

Environmental information is presented per API. Environmental hazard includes data on persistence (P), bioaccumulation (B), and ecotoxicity (T) of the API. Briefly, each of the characteristics is assigned a numeric value, 0–3, depending on its ability to resist degradation in the aquatic environment, its ability to accumulate in adipose tissue of aquatic organisms, and its ability to poison aquatic organisms respectively [13, 14, 23]. The sum of the individual values, 0–9, makes up the hazard score. A higher hazard score indicates a greater potential to harm the environment. The *T*-value in the hazard score can refer to either acute or chronic ecotoxicity. We use a precautionary approach; thus, APIs with incomplete data are assigned a higher value. In the database, lack of data is indicated with an asterisk [13, 14, 23].

When publicly available, a conclusion on risk is presented. The risk is based on a comparison between the predicted environmental concentration (PEC) of an API and the concentration expected to be safe to aquatic animals and plants (PNEC, predicted no effect concentration) [13, 24–26]. PEC is calculated from sales figures in Sweden or predicted use in Europe. The risk can be stated as “insignificant,” “low,” “moderate,” “high,” or “cannot be excluded.” “Cannot be excluded” is the term used when the pharmaceutical company has not provided enough data for a risk assessment. It is also stated if the API is exempted from risk assessment according to the guidelines of the Committee for Medicinal Products for Human Use (CHMP) [24].

The primary data sources for the database are the environmental information presented at fass.se and in the EPARs at EMA’s website. The environmental information at fass.se is based on data from the pharmaceutical companies [27]. These data are reviewed by the consultancy firm the Swedish Environmental Research Institute [27]. There are EPARs for medicinal products approved from 2006 when the CHMP guideline was implemented. In general, we accept the hazard and risk assessments provided by EMA and LIF, but we are aware of the previous studies showing deficiencies in the data provided [28, 29]. If environmental information is available both in EPARs and on fass.se, all is included in the database, and it is clearly stated on which data the overall assessment of hazard score and risk is based. The following aspects are considered when deciding this: the year and geographical area for the PEC-calculation, and the reliability and relevance of environmental fate and ecotoxicity studies.

Additional data from peer-reviewed literature are used when assessed as reliable and relevant. Comparative environmental assessments for medically comparable alternatives can be performed by external experts. These assessments may differ from the assessments available at fass.se and EMAs website since they can be based on measured environmental concentrations (MEC) and include risk of selection of antibiotic-resistant bacteria [14, 30]. When needed, academic experts are contacted for advice on how to interpret scientific studies and assessments, and the Swedish Medical Products Agency, EMA, and LIF are contacted for clarifications of available assessments.

### Assessing the use of the database

The use of the database *Pharmaceuticals and Environment* was assessed using Google Analytics. Database visits were analyzed for the period January to September 2018, and the corresponding period in 2017. In October 2018, the technical platform for the database and janusinfo.se was changed which is the reason why the period thereafter was not included in the analysis. The following analyses were performed for the Swedish and the English version of the database: total number of page views, top three countries for traffic by countries, and top ten APIs.

### Identifying lessons learned from developing and managing the database

During the years of developing and managing the database, different issues related to the function of the database as decision support have been discussed and handled. From these discussions, lessons learned have been phrased. The lessons concern presentation of information, access to data, handling discrepancies in data, comparison of data, and technical issues. This work has been done in collaboration between the person managing the database at Region Stockholm (H.R., pharmacist, PhD) and the database reference group (M-L.O., M.D. PhD; M.Å., environmental researcher, PhD; J.B., environmental strategist, PhD and Therese Olsen Sköldstam, environmental manager).

## Results

### Presentation of the database *Pharmaceuticals and Environment*

The database currently (29 March 2019) contains information about 851 APIs. Of these, 154 APIs are exempt and 175 APIs lack information about P, B, and T. For 379 APIs, the risk is assessed as “cannot be excluded,” i.e., data are missing or insufficient to calculate the risk. When updating the information in the database, we prioritize the APIs on the “Wise list” (the drug formulary of essential medicines for common diseases in Region Stockholm from the Drug and Therapeutics Committee). In addition, we also focus on other APIs widely used in Region Stockholm and APIs on Region Stockholm’s list of 25 environmentally harmful APIs. This list was developed as a part of Region Stockholm’s environmental program for the period 2017–2021, aiming to reduce negative environmental impact from APIs [31]. The expert panels of Region Stockholm Drug and Therapeutics Committee were involved in developing suggestions on how health care professionals can work to reduce environmental impact from the 25 APIs on the list. Their suggestions are included in the database.

**Fig. 1:**
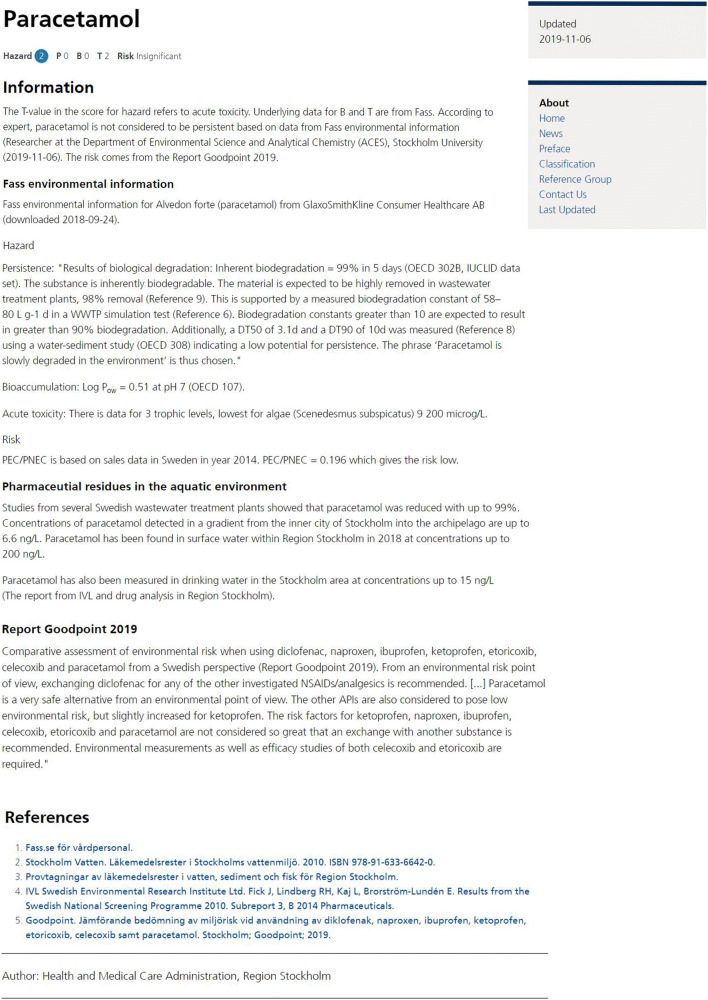
Information about paracetamol/acetaminophen from the database *Pharmaceuticals and Environment* at janusinfo.se [14]. P = persistence, B = bioaccumulation, and T = toxicity

Figure 1 shows how environmental information is presented in the database, using the API paracetamol/acetaminophen as an example [14]. Firstly, information about the hazard score and risk is provided. This is followed by information about the *T*-value which is based on acute study data and the reference for the underlying data for P, B, and T. Below that, data from the references are presented in more detail (from fass.se for “Alvedon forte” in this case). There is also information presented about detected levels in Swedish water environment, including drinking water, and results from a comparative assessment of environmental risk for diclofenac, naproxen, ibuprofen, ketoprofen, etoricoxib, celecoxib, and paracetamol/acetaminophen in Sweden. At the end, there is a reference list [14].

The API diclofenac provides an example of how extended information about an API in the database is presented [14]. Diclofenac is on Region Stockholm’s list of 25 environmentally harmful APIs. The database contains information about diclofenac previously being monitored within the EU Water Framework Directive and may be proposed as a priority substance when the European Parliament and the Council propose a revised directive for priority substances in the EU Water Framework Directive. It also states that the Swedish Agency for Marine and Water Management has included diclofenac among special pollutants in its regulation. One can also read that diclofenac has been detected in treated wastewater and surface water in Region Stockholm. Results from a comparative assessment of environmental risk of diclofenac, naproxen, ibuprofen, ketoprofen, etoricoxib, celecoxib, and paracetamol/acetaminophen in Sweden are presented. For the health care professionals, there is a reminder that diclofenac is not recommended on the “Wise list.” There is a concrete proposal that the health care professionals should review their recommendations to patients regarding over-the-counter analgesics. Finally there is a reference list [14].

### Use of the database

Metrics provided by Google Analytics show that the total number of page views for the Swedish version from January to September 2017 was 6138. For the corresponding period in 2018, it was 7341, i.e., an increase of approximately 20%. For the English version, the page views were 2095 in 2017 and 1931 in 2018, i.e., a decrease of approximately 8%. Table 1 presents the total number of page views, page views for the top 10 APIs, and traffic by countries for 2018, for the Swedish and the English version of the database.

**Table 1:** Use of the database during the period 2018-01-01—2018-09-30

Swedish version		English version	
Total number of page views, 7341		Total number of page views, 1931	
Top 10 APIs	Number of page views	Top 10 APIs	Number of page views
1.Diclofenac	832	1.Hydrochlorotiazide	71
2.Paracetamol	246	2.Diclofenac	51
3.Sertraline	227	3.Propofol	42
4.Tetracycline	216	4.Amoxicillin	26
5.Naproxen	169	5.Levonorgestrel	25
6.Ibuprofen	127	6.Cetirizine	24
7.Escitalopram	115	7.Estradiol	23
8.Felodipine	106	8.Ibuprofen	23
9.Ciprofloxacin	104	9.Amlodipine	22
10.Ethinylestradiol	88	10.Metoclopramide	22
Top 3 countries		Top 3 countries	
1.Sweden	5650	1.France	172
2.Finland	60	2.Canada	63
3.Denmark	21	3.UK	60

### Lessons learned from developing and managing the database

#### Information can be missing, incomplete, or removed

We have experienced that data provided in environmental risk assessments by EMA have been incomplete, e.g., lacked conclusion for bioaccumulation and/or persistence and the basis for calculating the environmental risk. When it is stated that CHMP had requested additional environmental information from a pharmaceutical company and the information could not be found at EMA’s website, we have contacted the Swedish Medical Products Agency or EMA for clarifications. In some cases, the requested information had not yet been submitted, even after several years. In other cases, data had been submitted and were available upon request, or submitted but not available upon request. Furthermore, environmental information is presented per product at EMA’s website, i.e., one must search through all approved medicinal products to find the available environmental information for a specific API.

Fass.se is the main source of information for medicinal products approved before 2006. However, for a considerable number of these substances, there is no environmental information available. When available, ecotoxicity studies primarily concerned acute endpoints as chronic studies were not required prior to the implementation of the CHMP guideline. At fass.se, the environmental information for medicinal products needs to be updated every three years, otherwise it is removed. It is our experience that the information is sometimes removed instead of being updated. In those cases, we make use of previously published information that we have downloaded, and it is made clear in the database.

#### A strategy for handling discrepancies in information is needed

The environmental information provided by EMA and LIF may differ for different medicinal products with the same API, this problem has been pointed out before [29, 32]. If there are different conclusions regarding an APIs ecotoxicity, persistence, or bioaccumulation, we present a worst-case scenario. If the PEC differs, we present a scenario with the most recent sales data for Sweden.

#### Deviations from guidelines are commented on

Deviations from the CHMP guideline and the guidance at fass.se have been found in the environmental risk assessments. For example, incorrect use of assessment factors for PNEC-calculations, or the use of ecotoxicity data with “greater than” (>) values. In those cases, the error is corrected, and/or a comment is provided in the database.

#### Different types of studies hamper comparisons of data

When Region Stockholm initiated the database, ecotoxicity studies primarily concerned acute endpoints, but since the CHMP guideline was implemented, chronic ecotoxicity data have become available [24]. This is a welcome development, but it can hamper the comparison of the ecotoxicity between APIs. In the database, it is specified for each API if the value for *T* in the hazard score refers to acute or chronic studies.

#### Following regulatory practice facilitates use

It is desirable that the terminology in the database is consistent with the terminology used within the regulatory framework. The hazard score was previously called PBT index but was renamed in 2018 as PBT/vPvB (Persistent, Bioaccumulative, and Toxic/very Persistent and very Bioaccumulative) and is the term used within the regulatory framework for Substances of Very High Concern [24]. Further, the hazard score was initially developed to guide decision-makers not familiar with risk assessment procedures in the comparison between APIs. However, it has proven to be a rather blunt instrument. An API with a higher score is not necessarily of greater environmental concern. Therefore, there is an ongoing discussion within the reference group to remove the hazard score from the database. This would be in line with current regulatory practice.

#### It is possible to generate and make use of new information

If there is no risk assessment in the EPAR even though enough data are provided to make the calculation, the risk is calculated following the guidelines [24, 33]. If ecotoxicity studies are missing at fass.se but have been published in previous environmental information at fass.se, the risk is calculated together with available sales data/PEC-value.

When deemed useful, an external expert has provided Region Stockholm with comparative environmental assessments on specific pharmaceutical groups, e.g., COX inhibitors, statins, and SSRIs. The purpose of these assessments is to clarify if any of the APIs within a pharmaceutical group is to be preferred from an environmental point of view. These investigations can be based on MEC and effect data for APIs, including risk of selection of antibiotic-resistant bacteria [14, 30].

Available exposure data have been supplemented with data from Region Stockholm’s screening program for APIs. It currently includes more than 100 APIs, measured primarily in the water environment [34]. In addition to the screening program carried out by Region Stockholm, other reports on pharmaceutical residues in water are also used as references for the database [35–37].

#### Transparency demands also apply to our work

In the previous printed brochure, risk and hazard scores including numeric values for the individual characteristics of P, B, and T were presented without the underlying data. To increase the transparency, underlying data for hazard assessment are now successively added to the information for individual APIs, including references for the information, see Fig. 1. Furthermore, the underlying data for the exposure assessment, and consequently the conclusion on risk, are included. This is important since the exposure assessment for different APIs may be based on PEC (using either expected use or sales data from different years), or on MEC. The increased transparency facilitates critical evaluation of the assessment and comparison between APIs.

#### A user-friendly technical platform is a crucial key

The transfer of the environmental information to a database using a Content Management System offers benefits to users. More comprehensive information can be presented, and a reference list is included. The content is kept more up to date as compared with the printed folder. For the editor, the transfer from the Excel-based system facilitates handling of more information.

## Discussion

Decision support on environmental effects for pharmaceuticals is needed, both in the private and public sector, to address the potential problems related to production and use of pharmaceuticals. It is our opinion that decision support should primarily be provided by regulatory agencies, on national (e.g., the Swedish Medical Products Agency) or EU-level (e.g., the EMA or the European Commission). There are two reasons for this: their presumed objectivity and their access to public and confidential information. Currently, the Swedish Medical Products Agency does not provide any decision support and, as shown in this paper, the information provided by EMA is not always sufficient to serve as decision support.

The development of other databases also indicates that the decision support provided has not been sufficient. It is our interpretation that this absence of adequate decision support relates to that environmental effects from pharmaceuticals is not a field of particular interest to decision-makers. This can be seen both when comparing the management of pharmaceuticals with other chemicals and comparing “environmental side effects” with medical side effects of pharmaceuticals. We think this conclusion is supported by several observations. The first legal requirement for environmental risk assessment of pharmaceuticals was established relatively late [38] and the requirements are low in comparison with those for other chemical groups. This is illustrated by the fact that the requirements only apply to medicinal products seeking marketing authorization after the legislation was brought into force. Also, the legal demands on companies to provide the requested environmental information appear to lack power as environmental information is still missing several years later. Other important processes related to environmental effects from pharmaceuticals have also been delayed or ignored. The EU’s “Strategic approach to pharmaceuticals in the environment” was delayed by several years, and no actions have been taken to deal with the severe emissions from production of pharmaceuticals for the EU market [39].

Another indication of the indifference is that environmental effects are not included in the monitoring of the safety of pharmaceuticals that is carried out by manufacturers and regulatory agencies throughout their life cycle. EU law requires marketing authorization holders, national competent authorities, and EMA to operate a pharmacovigilance system, but that does not include environmental aspects. Just as there can be new studies published on medical effects and side effects of pharmaceuticals after approval, new environmental studies could alter the assessments.

The database *Pharmaceuticals and Environment* at janusinfo.se presents compiled, comprehensive, and easily accessible environmental information about APIs. This information can be used when communicating about environmental risks, and as decision support when assessing whether it would be favorable, from an environmental point of view, to substitute one API for another. The content in the database has a Stockholm and Sweden perspective in terms of use (sales data) and MEC but hazard assessments are primarily based on non-site-specific tests.

Other medical knowledge databases within Region Stockholm, e.g., the databases *Drugs and Birth Defects*, and *Interactions* [40, 41], are designed to be decision supports in every-day clinical work. In contrast, the database *Pharmaceuticals and Environment* offers the Stockholm Drug and Therapeutics Committee, and other Swedish Drug and Therapeutics Committees, a possibility to include environmental aspects when recommending pharmaceuticals for health care providers [42]. Many Drug and Therapeutics Committees in Sweden include environmental aspects when making pharmacotherapeutic recommendations. These recommendations help physicians and other prescribers to make good environmental choices without compromising medical effectiveness and safety when prescribing pharmaceuticals [42]. Since the database contains suggestions on how to reduce the environmental impact from particularly environmentally harmful APIs, the database can also be used as a management tool by health care providers. Also, researchers and wastewater treatment operators can make use of the compiled environmental information. To increase our understanding of the current use and how the database could be further developed, it would be interesting to perform a survey among users.

From the lessons learned, we conclude that one major problem is lack of environmental information for a majority of APIs, not least among the medicinal products approved before 2006 [24]. This has also been noted by the European Commission and others [19, 39, 43]. For medicinal products approved before 2006, the database is dependent on the information available through fass.se and the peer-reviewed literature. A drawback with older information is that it mainly reflects the acute ecotoxicity of the APIs. In addition, since it is optional for companies to publish information at fass.se, medicinal products may lack environmental information completely. Also, environmental information that has been published can be withdrawn, which makes fass.se a precarious source of information. It has been estimated that 5–10% of medicines in use might pose an appreciable risk to the environment [32, 44]. A pragmatic way to identify these substances for further environmental assessments could be to apply a prioritization approach [44].

From an environmental point of view, it is desirable to find medically comparable alternatives that are less environmentally harmful. In decision-making, it is not enough to know that one API has a negative environmental impact if data are missing for the alternatives. The assessment of whether substituting one API for another is environmentally beneficial or harmful also poses a challenge.

For the environmental risk assessments presented by EMA and LIF, it would be desirable that the complete environmental risk assessment, including underlying studies, is made publicly available and easily accessible. At present, there is no regulation regarding which information that needs to be made public in the EPARs. CHMP’s interpretation of data could also be more perspicuous; there are for example several EPARs communicating test results for persistence without a conclusion. It is desirable that CHMP develops a process to ensure that all requested information in the environmental risk assessments is submitted by the manufacturers. Furthermore, it would be desirable that environmental data are presented in a compiled assessment per API and that all the information is easily accessible at EMA’s website. A change in consumption of a medicinal product can alter the calculated risk, and new knowledge could affect the conclusions in a previously performed environmental risk assessment. It would therefore be valuable if the environmental information for APIs is updated at regular intervals, which is standard procedure in regulatory frameworks for other chemicals [38]. However, since the benefit/risk assessment for human medicinal products at present does not include environmental effects, an update of the environmental risk assessment is not required for renewals of marketing authorizations [24].

We welcome the proposal for a revised EMA guideline [45]. The proposal includes a tailored risk assessment for APIs with specific classifications (e.g., endocrine active substances, antibiotic substances) and further tests, e.g., the estimation of the exposure of predators to pharmaceuticals through the food chain (“secondary poisoning”), as well as directly through the environment. If the guideline were to be updated with the proposed changes, it would improve the information in the environmental risk assessments, and hence in the database.

In the absence of an adequate decision support produced by the regulatory authorities, we find the database *Pharmaceuticals and Environment* to be a useful decision support for Swedish Drug and Therapeutics Committees and health care providers, and it is our belief that the information can be valuable also in other settings.

## Data Availability

The information in the database is available in Swedish and English at janusinfo.se.
